# Association of traffic-related hazardous air pollutants and cervical dysplasia in an urban multiethnic population: a cross-sectional study

**DOI:** 10.1186/1476-069X-13-52

**Published:** 2014-06-13

**Authors:** Michael E Scheurer, Heather E Danysh, Michele Follen, Philip J Lupo

**Affiliations:** 1 Department of Pediatrics, Section of Hematology-Oncology and Dan L. Duncan Cancer Center, Baylor College of Medicine, Houston, Texas; 2 Department of Obstetrics and Gynecology, Paul L. Foster School of Medicine, Texas Tech University Health Sciences Center, El Paso, Texas

**Keywords:** Benzene, Cervical dysplasia, Diesel particulate matter, Hazardous air pollutants, Polycyclic aromatic hydrocarbons

## Abstract

**Background:**

Human papillomavirus (HPV) infection is a necessary cause in the development of cervical cancer; however, not all women infected with HPV develop cervical cancer indicating that other risk factors are involved. Our objective was to determine the association between exposure to ambient levels of common traffic-related air toxics and cervical dysplasia, a precursor lesion for cervical cancer.

**Methods:**

The study sample consisted of women enrolled in a Phase II clinical trial to evaluate diagnostic techniques for cervical disease in Houston, Texas. The current assessment is a secondary data analysis in which cases were defined as women diagnosed with cervical dysplasia, while those without cervical dysplasia served as controls. Residential census tract-level estimates of ambient benzene, diesel particulate matter (DPM), and polycyclic aromatic hydrocarbons (PAHs) were used to assess exposure. Census tract-level pollutant estimates were obtained from the United States Environmental Protection Agency. Multivariable logistic regression was used to estimate prevalence odds ratios (aOR) and 95% confidence intervals (CI) adjusted for age, race/ethnicity, education, smoking status, and HPV status.

**Results:**

Women in the highest residential exposure categories for benzene and DPM had an increased prevalence of cervical dysplasia compared to the lowest exposure category (Benzene: aOR [95% CI] for high exposure = 1.97[1.07-3.62], very high exposure = 2.30[1.19-4.46]. DPM: aOR [95% CI] for high exposure = 2.83[1.55-5.16], very high exposure = 2.10[1.07-4.11]). Similarly, women with high residential exposure to PAHs had an increased prevalence of cervical dysplasia (aOR [95% CI] = 2.46[1.35-4.48]). The highest PAH exposure category was also positively associated with cervical dysplasia prevalence but was not statistically significant. Assessment of the combined effect of HAP exposure indicates that exposure to high levels of more than one HAP is positively associated with cervical dysplasia prevalence (p for trend = 0.004).

**Conclusions:**

Traffic-related HAPs, such as benzene, DPM, and PAHs, are not as well-regulated and monitored as criteria air pollutants (e.g., ozone), underscoring the need for studies evaluating the role of these toxicants on disease risk. Our results suggest that exposure to traffic-related air toxics may increase cervical dysplasia prevalence.

## Background

Cervical cancer is the third most common cancer and the fourth leading cause of cancer-related mortality among women worldwide [1]. The majority (>85%) of cervical cancer cases and deaths occur in the developing world [1], largely due to limited screening programs which allow for the detection of cervical dysplasia (precancerous lesions) and early stage cervical cancer [2-4]. Infection with human papillomavirus (HPV) has been established as a necessary cause in the development of cervical cancer; however, most women infected with HPV do not develop cervical cancer [5,6]. Lifestyle factors, such as sexual behavior and smoking cigarettes [7,8], use of oral contraceptives [9,10], high parity [10,11], as well as co-infections, such as human immunodeficiency virus infection [12], are associated with the development of cervical cancer. Taken together these findings suggest that genetic and/or environmental factors may play a role in the development of this malignancy [13].

Air pollution is largely composed of automobile emissions, a complex mixture of compounds many of which are known to adversely affect human health. Benzene, polycyclic aromatic hydrocarbons (PAHs), and diesel particulate matter (DPM) are components of automobile emissions and have been designated by the United States Environmental Protection Agency (U.S. EPA) as hazardous air pollutants (HAPs) [14]. HAPs are toxic substances known or suspected to be carcinogenic. In fact, the International Agency for Research on Cancer has classified benzene and DPM as carcinogenic compounds, and many PAHs as possible, probable, or known carcinogens [15-17]. Some studies have suggested that exposure to traffic-related air pollutants, such as benzene, PAHs, and DPM, is associated with an increased risk for several types of cancer, including lung cancer [18,19], brain cancer [20,21], and leukemia [22-24]. The carcinogenic impact of exposure to traffic-related air pollution is an ever-increasing public health concern as urbanization continues to rise, and a greater proportion of the population is exposed to higher levels of HAPs.

Exposure to traffic-related HAPs may play a role in the development of cervical cancer. Occupational exposure to diesel engine exhaust has previously been shown to be associated with an increased risk of cervical cancer among women [25]. In addition, a recent study found an increased risk of cervical cancer in Danish women with higher residential concentration levels of nitrogen oxides (NO_x_), a component of automobile engine emissions [21]. The objective of the current study is to evaluate the association between levels of traffic-related HAPs and cervical dysplasia, a precursor lesion for cervical cancer, in a multi-ethnic sample of women receiving cervical cancer screening and diagnostic services in Houston, Texas.

## Methods

### Study population

The study population consisted of women attending the colposcopy clinics at The University of Texas MD Anderson Cancer Center and Lyndon B. Johnson General Hospital in Houston, Texas, between 2000 and 2004, and enrolled in a multi-center Phase II clinical trial (registered at http://www.ClinicalTrials.gov as NCT00511615) to evaluate fluorescence and reflectance spectroscopy for diagnosing cervical disease. The detailed data collection methods for this trial have been previously described [26]. Briefly, women had to be 18 years of age or older, have no history of hysterectomy, and not be pregnant to be eligible for enrollment in the parent trial. Of those who were eligible and enrolled in the parent trial, only women who had available information on HPV infection status and residence (i.e., address) at the time of diagnosis were eligible for our study, a secondary data analysis using data collected during the parent trial. After applying all eligibility criteria, 736 women were included in the current assessment. Cases (n = 173) included women who were diagnosed with cervical intraepithelial neoplasia (CIN) I, CIN II, or CIN III. Women who had a normal Papanicolaou test result (i.e., not diagnosed with cervical dysplasia or cervical cancer) served as control subjects (n = 563). Details on the laboratory methods used to test for HPV status as well as those used to confirm cervical dysplasia disease status have been previously described [26]. Epidemiologic data were obtained from a risk factor interview conducted with each patient at the time of enrollment on the parent trial. Women received the standard treatment according to their colposcopy results. The Institutional Review Boards at the University of Texas MD Anderson Cancer Center and Harris Health System approved the protocol for the parent trial. Informed consent was obtained from all subjects.

### Ambient air pollutant concentration levels

Annual concentration estimates of benzene, DPM, and PAHs were obtained for each census tract from the U.S. EPA’s 1999 Assessment System for Population Exposure Nationwide (ASPEN). ASPEN is a computer simulation model used in the National-Scale Air Toxics Assessment conducted by the U.S. EPA [27]. The ASPEN model was derived from the U.S. EPA’s Industrial Source Complex Long Term model designed to model air pollutant dispersion. To generate annual pollutant concentration estimates, ASPEN uses information on meteorological conditions, the location and height of pollutant release, rate of release, and pollutant deposition, reactive decay, and transformation properties. Other epidemiological studies have used pollutant concentration estimates from ASPEN to evaluate the effect of HAPs on disease risk [23,28,29].

Residential pollutant levels were determined based on the ASPEN estimates for the census tract of the subject’s residence at the time of the clinic visit. Addresses were geocoded and mapped to determine residential census tracts using ArcGIS software (Esri, Redlands, California). Benzene, DPM, and PAH levels from ASPEN were categorized using the distribution of the residential HAP levels among the controls: low or reference exposure (<25th percentile), medium exposure (25th-74th percentiles), high exposure (75th-89th percentiles), and very high exposure (≥90th percentile). In addition, a HAP composite score was created to assess the combined effect of exposures to multiple HAPs on cervical dysplasia. To create the HAP composite score, each exposure category of each HAP was assigned a score: 0 = low, 1 = medium, 2 = high, and 3 = very high. The scores from the three HAPs were then summed for each subject based on their residential census tract in order to generate the overall HAP composite score, which could range from 0–9.

### Statistical analysis

Descriptive statistics were generated to characterize the demographic variables among the case and control groups, which included computing means and standard deviations (SD) for continuous variables and frequency distributions for categorical variables. Potential differences between the case and control groups in the distribution of each of the demographic variables were tested for statistical significance using *t* tests for continuous variables and *χ*^2^ tests for categorical variables. Correlations between levels of benzene, DPM, and PAHs were determined using Spearman’s rank correlation. We used multivariable logistic regression to assess the associations between HAP levels and cervical dysplasia. Adjusted prevalence odds ratios (aOR) and 95% confidence intervals (CI) were estimated for each association. Covariates were selected *a priori* and included age, race/ethnicity, years of completed education, HPV infection status [30,31], and smoking status. All multivariable regression models were adjusted for these covariates. Univariable logistic regression analyses were conducted to evaluate the associate between cervical dysplasia and demographic variables, including age, race/ethnicity, smoking status, and completed years of education, variables which also serve as covariates in the adjusted analyses. These results were included in Additional file 1: Table S1.

## Results

There were no statistically significant differences between the case and control groups in regard to racial makeup, completed years of education, smoking status, HPV infection status, and the screening institution (Table 1). Approximately half of the sample was non-Hispanic white (52.5%), with a quarter being Hispanic (24.2%) and fewer being non-Hispanic black (16.0%). This racial/ethnic distribution approximates the distribution of the population in the Houston area. The mean (SD) completed years of education was 13.3 (3.3) years. Approximately one-third of the sample (32.5%) had a history of smoking. Most of the subjects (both cases and controls) were positive for HPV infection (73.0%); 27.0% were HPV negative. The majority of the sample (84.4%) was screened at the colposcopy clinic at the University of Texas MD Anderson Cancer Center, while the remainder (15.6%) was screened at the Lyndon B. Johnson General Hospital. Cases were younger (*p* < 0.001) and more likely to have never been married (*p* = 0.04) compared to controls. Cases primarily had CIN I (81.5%) while the remainder of the cases had moderate to severe dysplasia (18.5%). All controls were confirmed to not have cervical dysplasia.

**Table 1:** Characteristics of cervical dysplasia cases and controls from colposcopy clinics in Houston, Texas, 2000-2004

**Characteristic**	**Cases (*n*** **= 173)**	**Controls (*n*** **= 563)**	**P-value**
Age in years, mean ± SD	37.0 ± 11.0	43.5 ± 11.8	<0.001
Race/ethnicity, *n* (%)			
Non-Hispanic White	90 (52.0)	296 (52.6)	0.180
Non-Hispanic Black	36 (20.8)	85 (15.1)	
Hispanic	39 (22.5)	137 (24.3)	
Other	8 (4.6)	45 (8.0)	
Education in years, mean ± SD	13.1 ± 1.98	13.4 ± 3.57	0.376
Marital status, *n* (%)			
Never	45 (26.0)	102 (18.2)	0.038
Married/partnered	85 (49.1)	331 (58.9)	
Divorced/widowed/separated	43 (24.9)	129 (23.0)	
Smoking status, *n* (%)			
Never	108 (62.4)	389 (69.1)	0.101
Ever	65 (37.6)	174 (30.9)	
HPV status, *n* (%)			
Positive	129 (74.6)	408 (72.5)	0.587
Negative	44 (25.4)	155 (27.5)	
Histology, *n* (%)			
CIN I (mild dysplasia)	141 (81.5)		
CIN II (moderate dysplasia)	19 (11.0)		
CIN III (severe dysplasia)	13 (7.5)		
Clinic, *n* (%)			
MD Anderson Cancer Center	141 (81.0)	481 (85.4)	0.153
Lyndon B. Johnson General Hospital	33 (19.0)	82 (18.6)	

*Abbreviations:* SD, standard deviation; HPV, human papillomavirus; CIN, cervical intraepithelial neoplasia.

The distributions of selected census tract-level HAP concentration levels among the control group are presented in Table 2. The mean benzene exposure level among the controls was 1.917 μg/m^3^, with the lowest (<25^th^ percentile) and highest (≥90^th^ percentile) exposure categories representing those living in census tracts with an estimated average benzene level <1.415 μg/m^3^ and ≥2.977 μg/m^3^, respectively. Similarly, the mean level of DPM and PAHs among the controls was 2.015 μg/m^3^ and 0.011 μg/m^3^, respectively. The lowest and highest exposure categories included those living in census tracts with an estimated average DPM level <1.413 μg/m^3^ and ≥2.794 μg/m^3^, respectively. For PAHs, the lowest exposure category was <0.007 μg/m^3^ and the highest exposure category was ≥0.018 μg/m^3^.

**Table 2:** Distributions of hazardous air pollutants

**Pollutant (μg/m**^**3**^)*	**Mean**	**25**^**th **^**percentile**	**50**^**th **^**percentile**	**75**^**th **^**percentile**	**90**^**th **^**percentile**
Benzene	1.917	1.415	1.760	2.261	2.977
DPM	2.015	1.413	1.724	2.175	2.794
PAHs	0.011	0.007	0.009	0.013	0.018

*Abbreviations:* DPM, diesel particulate matter; PAHs, polycyclic aromatic hydrocarbons.

*All concentrations are presented as μg/m^3^.

**Figure 1:**
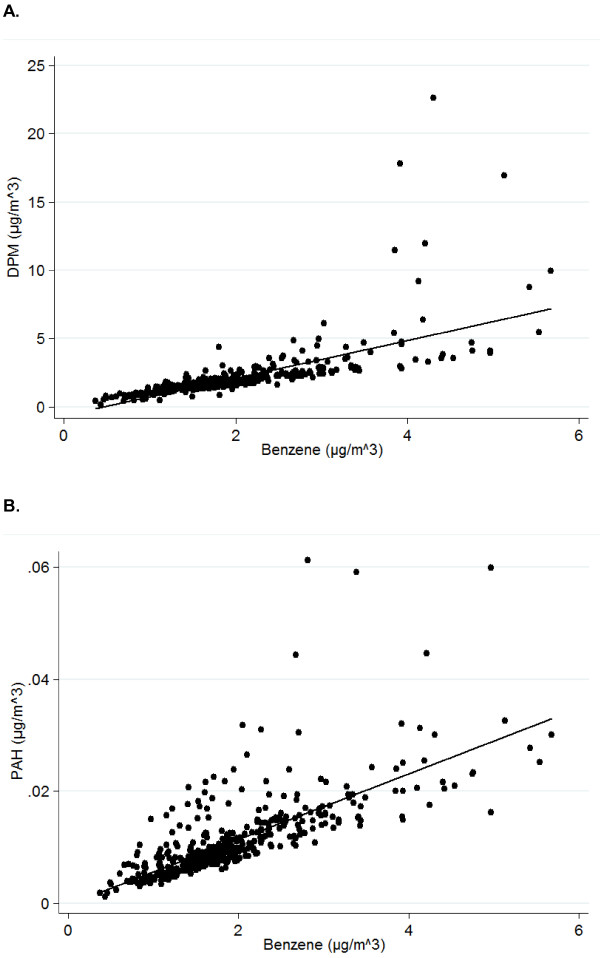
**Correlations between traffic-related hazardous air pollutant levels.** Scatterplots of **(A)** diesel particulate matter (DPM) and benzene, **(B)** polycyclic aromatic hydrocarbons (PAHs) and benzene from the 1999 United States’ Environmental Protection Agency’s (U.S. EPA) Assessment System for Population Exposure Nationwide’s (ASPEN) model for Texas census tracts included in the current assessment.

Scatterplots of benzene and each of the other HAPs (i.e., DPM and PAHs) are presented in Figure 1 to assess correlations between the pollutant levels. Overall, benzene levels are highly correlated with levels of DPM (ρ = 0.91, *p* < 0.0001) and PAHs (ρ = 0.80, *p* < 0.0001). However, these correlations were not as strong when restricted to those in the “very high” exposure category. Specifically, when restricting the correlation analysis to those in the very high benzene exposure category (*n* = 83) the correlations with DPM (ρ = 0.66, *p* < 0.0001) and PAHs (ρ = 0.65, *p* < 0.0001) are attenuated.

The aORs and 95% CIs of the associations between residential exposure to benzene, DPM and PAH's and the prevalence of cervical dysplasia are presented in Table 3. Residential exposure to benzene was associated with cervical dysplasia (aOR medium exposure = 1.39, 95% CI: 0.83-2.32; aOR high exposure = 1.97, 95% CI: 1.07-3.62; and aOR very high exposure = 2.30, 95% CI: 1.19-4.46) after adjusting for age, race/ethnicity, years of education, HPV infection status, and smoking. We also observed a trend with increasing exposure (*p* for trend = 0.005). Residential exposure to DPM is positively associated with cervical dysplasia when comparing high and very high exposure to low exposure (aOR for high exposure = 2.83, 95% CI: 1.55-5.16; aOR for very high exposure = 2.10, 95% CI: 1.07-4.11). Compared to low exposure, women living in census tracts with high levels of PAHs had an increased prevalence of cervical dysplasia (aOR = 2.46, 95% CI: 1.35-4.48). The effect estimates for medium or very high exposure to PAHs suggest an increased prevalence of cervical dysplasia; however, these associations are not statistically significant. When assessing the combined effect of HAP exposure as defined using the HAP composite score, there is a positive association with cervical dysplasia prevalence when comparing women with a higher (score of 3+) HAP composite score to those with a low composite score (score <3) (aOR for medium score = 1.78, 95% CI: 1.11-2.84; aOR for high score = 2.30, 95% CI: 1.28-4.13). All regression analyses were repeated on a restricted sample that excluded cases with mild dysplasia, which yielded similar results (data not shown), and therefore we retained the grouping of mild and progressive cervical disease in our final models [30,32]. Moreover, regression analyses were repeated stratifying the sample on HPV infection status, and the overall results were similar regardless of HPV status (data not shown).

**Table 3:** Associations between selected traffic-related hazardous air pollutants and cervical dysplasia

**Pollutant**	Prevalence aOR* [95% CI]
Benzene	
Low (<25^th^ percentile)	1.00
Medium (25^th^-74^th^ percentile)	1.39 [0.83, 2.32]
High (75^th^-89^th^ percentile)	1.97 [1.07, 3.62]
Very high (≥90^th^ percentile)	2.30 [1.19, 4.46]
	P for trend = 0.005
DPM	
Low (<25^th^ percentile)	1.00
Medium (25^th^-74^th^ percentile)	1.41 [0.84, 2.37]
High (75^th^-89^th^ percentile)	2.83 [1.55, 5.16]
Very high (≥90^th^ percentile)	2.10 [1.07, 4.11]
	P for trend = 0.002
PAHs	
Low (<25^th^ percentile)	1.00
Medium (25^th^-74^th^ percentile)	1.30 [0.77, 2.20]
High (75^th^-89^th^ percentile)	2.46 [1.35, 4.48]
Very high (≥90^th^ percentile)	1.88 [0.94, 3.73]
	P for trend = 0.006
HAP composite	
Low (score 0–2)	1.00
Medium (score 3–6)	1.78 [1.11, 2.84]
High (score 7–9)	2.30 [1.28, 4.13]
	P for trend = 0.004

*Abbreviations:* aOR, adjusted odds ratio; CI, confidence interval; DPM, diesel particulate matter; PAHs, polycyclic aromatic hydrocarbons; HAP, hazardous air pollutant.

*Odds ratios adjusted for age, race/ethnicity, education, smoking status, and status of human papilloma virus (HPV) infection.

## Discussion

In one of the first studies of its kind, our results indicate traffic-related HAPs are associated with cervical dysplasia. Specifically, women living in census tracts with high levels of benzene, DPM, or PAHs were approximately two to three times more likely to be diagnosed with cervical dysplasia compared to women living in census tracts with relatively low pollutant levels. We also observed a statistically significant dose–response relationship between each pollutant and cervical dysplasia prevalence (*p* for trend ≤ 0.006 for benzene, PAHs, and DPM). These findings are notable as cervical dysplasia in conjunction with chronic HPV infection is a precursor of cervical cancer. In fact, 18.5% of our cases had medium to severe dysplasia (CIN II or higher), lesions associated with an increased risk of progression to cervical cancer, and the majority of the women in our sample were positive for HPV infection.

While previous studies have not evaluated the role of air pollution on the risk of cervical dysplasia, our findings are in keeping with previous reports of an increased risk of cervical cancer after exposure to traffic-related pollutants and HAPs [21,25]. In a recent assessment by Raaschou-Nielsen *et al.* using data from the Danish Diet Cancer and Health cohort, residential exposure to NO_x_ was associated with an increased risk of cervical cancer (incidence rate ratio = 2.45, 95% CI: 1.01-5.93) [21]. Another study conducted in Sweden indicated that occupational exposure to diesel engine emissions was associated with an increased incidence of cervical cancer (standardized incidence ratio = 1.48, 95% CI: 1.17-1.84) [25]. Our study builds upon these assessments by including a multi-ethnic population and evaluating cervical dysplasia rather than cervical cancer. In addition, there is considerable variation in HAP concentration levels between the HAP exposure categories defined in our analysis, which also includes HAP levels positively associated with adverse health risks [33].

Our findings are consistent with the role of cigarette smoking on cervical cancer risk. A large body of epidemiological evidence supports the association between cigarette smoking and cervical cancer risk [34-36]. In fact, when assessing smoking as a predictor of cervical cancer among women who have a confirmed HPV-positive status, those who were current or ever smokers had a two times greater risk of developing cervical cancer compared to never smokers (OR = 2.17, 95% CI: 1.46-3.22) [8]. Even passive exposure to cigarette smoke (i.e., second-hand smoke) may increase the risk of cervical cancer [37]. These previously reported associations are of note, as cigarette smoke contains carcinogenic compounds similar to those found in automobile emissions (e.g., benzene and PAHs), and, consequently, the effect of exposure to traffic-related air pollutants may be analogous to the effect of smoking on cervical cancer risk.

While the mechanism underlying the association between these pollutants and cervical dysplasia is unclear, there are certain biological pathways that may lead to dysplasia after exposure to HAPs. For instance, these HAPs may have genotoxic effects on the cervix. This is supported by evidence of high levels of PAHs in cervical mucus and cervical tissue among smokers [38]. PAH-DNA adducts have also been found in the cervical tissue of smokers [39]. These adducts have genotoxic properties, which may lead to cancer. It is unknown if benzene or DPM has been identified in cervical tissue, however, these compounds are also known to have genotoxic effects [15,16]. Another potential mechanism may be related to oxidative stress, as these pollutants have been shown to form reactive oxygen species, leading to DNA damage [40]. These pollutants may also play a role in an altered immune environment leading to a state of chronic inflammation [41,42]. Ultimately, it is suspected that these pollutants may lead to a state of DNA damage and/or inflammation, which may facilitate viral integration, resulting in enhanced cervical carcinogenesis. For instance, some evidence suggests that PAHs may actually play a role in the proliferation and persistence of HPV [43].

There are several limitations to consider when interpreting the results presented in this report. First, the cross-sectional study design and the inclusion of prevalent cases of cervical dysplasia make it difficult to assess temporality in terms of the timing of exposure and the development of disease. However, it is not uncommon to evaluate prevalent cases when assessing an asymptomatic condition that requires an invasive screening technique for detection [26]. Second, ASPEN yields area-based pollutant concentration estimates and therefore exposure misclassification is a possibility. Although we used an area-based exposure measure there are few sources of population-based exposure assessments of HAPs. Additionally, ASPEN pollutant concentration estimates have been used extensively in other assessments of HAPs and adverse health outcomes [23,28,29]. Lastly, it is not clear if the women in this assessment later developed more progressive cervical disease or cervical cancer; however, cervical dysplasia is an important precursor to cervical cancer and therefore it is important to understand risk factors for cervical dysplasia.

To our knowledge, this study is the first to evaluate the association of air pollution exposure and predictors of cervical dysplasia in a racially diverse sample of women. A growing body of evidence shows that there are disparities in air pollution exposure among racial minorities and those with a low socioeconomic status [44-46]. Racial minorities are more likely to live in urban areas with higher concentrations of traffic-related air pollution, therefore, increasing the risk of these groups to related health effects, such as cervical dysplasia. In addition, Hispanic and black women are less likely to receive Pap screening services, have a higher incidence of cervical cancer, and have poorer survival after a cervical cancer diagnosis compared to white women in the U.S. [47-51]. The vulnerability of racial and ethnic minority groups to air pollution exposure in combination with lower adherence to screening is a pressing public health concern.

The results from this study have pertinent implications for health in the global context. Cervical cancer incidence and mortality is highest in the developing world, specifically in Africa and Central and South America [1,52]. This is largely due to the high prevalence of HPV infection in conjunction with limited access to screening services to detect precancerous lesions and early stage cervical cancer, as well as the lack of effective dissemination of the HPV vaccine and targeted vaccine delivery programs [4,52,53]. Moreover, due to increasing urbanization, specifically in Latin American cities, a larger portion of the population living in developing regions is being exposed to high levels of air pollution largely resulting from rapidly growing roadway and traffic density in urban areas [54,55]. The increased prevalence of exposure to traffic-related HAPs in these vulnerable populations may lead to an even higher incidence of cervical cancer.

## Conclusions

This is the first study to evaluate the association between exposure to traffic-related HAPs and cervical dysplasia prevalence in a multi-ethnic sample of women. Our results suggest that women with high residential exposure to benzene, DPM, or PAHs have an increased prevalence of cervical dysplasia compared to women with relatively low exposure to these pollutants. Our findings highlight the need to continue to identify novel risk factors that contribute to cervical disease in conjunction with HPV infection. In addition, our findings may also be important in future cervical cancer prevention efforts as public health strategies are directed toward the detection, management, and prevention of cervical dysplasia.

## Abbreviations

aOR: Adjusted prevalence odds ratio; ASPEN: Assessment System for Population Exposure Nationwide; CI: Confidence interval; CIN: Cervical intraepithelial neoplasia; DNA: Deoxyribonucleic acid; DPM: Diesel particulate matter; HAPs: Hazardous air pollutants; HPV: Human papillomavirus; NO_x_: Nitrogen oxides; PAHs: Polycyclic aromatic hydrocarbons; SD: Standard deviation; U.S. EPA: United States Environmental Protection Agency.

## Competing interests

The authors declare that they have no competing interests.

## Authors’ contributions

MES and PJL conceived and designed the study. PJL carried out the primary data analysis with the assistance of HED. MES and HED drafted the initial manuscript. MF was the PI of the parent study and provided input regarding study design and the assessment of cervical dysplasia. All authors contributed to revisions of the manuscript and read and approved the final manuscript.

## Supplementary Material

Additional file 1: Table S1 Associations between demographic variables and cervical dysplasia: Univariable analyses. Click here for file
