# Supplementary material for: Association of traffic-related hazardous air pollutants and cervical dysplasia in an urban multiethnic population: a cross-sectional study
Source: Environ Health. 2014 Jun 13;13:52. doi: 10.1186/1476-069X-13-52 (PMC4063240; doi:10.1186/1476-069X-13-52)
Supplement: Additional file 1: Table S1 — Associations between demographic variables and cervical dysplasia: Univariable analyses. [file 1476-069X-13-52-S1.docx]

**Supplemental Table. Associations between demographic variables and cervical dysplasia: Univariable analyses**

| **Characteristic** | **Prevalence OR [95% CI]** |
| --- | --- |
| Age (years) | 0.95 [0.94, 0.97] |
| Race/ethnicity |  |
| Non-Hispanic White | 1.00 [Reference] |
| Non-Hispanic Black | 1.38 [0.87, 2.17] |
| Hispanic | 0.93 [0.60, 1.42] |
| Other | 0.58 [0.26, 1.27] |
| Education (years) | 0.96 [0.88, 1.05] |
| Smoking status |  |
| Ever | 1.00 [Reference] |
| Never | 1.37 [0.96, 1.95] |
